# Similarity maps - a visualization strategy for molecular fingerprints and machine-learning methods

**DOI:** 10.1186/1758-2946-5-43

**Published:** 2013-09-24

**Authors:** Sereina Riniker, Gregory A Landrum

**Affiliations:** 1 Novartis Institutes for BioMedical Research, Basel, Switzerland

**Keywords:** Visualization, Machine-learning, Similarity, Fingerprints

## Abstract

Fingerprint similarity is a common method for comparing chemical structures. Similarity is an appealing approach because, with many fingerprint types, it provides intuitive results: a chemist looking at two molecules can understand why they have been determined to be similar. This transparency is partially lost with the fuzzier similarity methods that are often used for scaffold hopping and tends to vanish completely when molecular fingerprints are used as inputs to machine-learning (ML) models. Here we present similarity maps, a straightforward and general strategy to visualize the atomic contributions to the similarity between two molecules or the predicted probability of a ML model. We show the application of similarity maps to a set of dopamine D3 receptor ligands using atom-pair and circular fingerprints as well as two popular ML methods: random forests and naïve Bayes. An open-source implementation of the method is provided.

## Background

Chemical structures are often represented by molecular fingerprints where structural features are converted to either bits in a bit vector or counts in a count vector. This abstract representation allows the computationally efficient handling and comparison of chemical structures. Using such fingerprints, the similarity between two molecules can be calculated in a straightforward manner with simple similarity metrics such as Tanimoto [1], Dice [2], and so on. However, depending on the descriptors used to generate the fingerprints, the interpretation of the resulting similarity may not be trivial. This problem worsens when machine-learning (ML) models are trained to predict the activity (or other properties) of new compounds: ML models often appear as complete "black boxes" that just output numeric predictions to their users. Though these predictions can be quite accurate, it has been shown that supplementing numeric predictions with additional information from the model can improve the ability of both expert and non-expert users to work with the results [3]. This provides substantial motivation for the development of strategies to visualize the parts of a molecule contributing to a similarity value or model prediction.

Few visualization approaches for such models are described in the literature. An early example is the visualization of a modal fingerprint [4,5], which contains all bits which are present in 50 - 100% of the molecules of a training set. The atoms are colored based on the similarity to the modal fingerprint, i.e. how many of the bits set by the atom are present in the modal fingerprint. Franke *et al.*[6] visualized the importance of three-point pharmacophores (3PP) obtained from a trained support vector machine (SVM) model by placing differently sized spheres at the centre of the substructure leading to a 3PP. The importance of each 3PP was calculated based on the difference of SVM prediction for a molecule when this 3PP is removed. The interpretation of linear SVM models was also the goal of the heat map coloring scheme developed by Rosenbaum *et al.*[7]. The SVM model was trained using ECFP fingerprints and the authors focussed solely on the coloring of bonds. The coloring was based on the weights obtained from the SVM model, where the final weight of a bond is the normalized sum of the weights of the fingerprints features containing this bond. The color scheme was chosen such that red corresponds to the negative class and green to the positive class with orange as zero. Another approach is the Glowing Molecule visualization which has been used to show the regions of a molecule which may have the most influence on ADME and physicochemical properties [8,9]. A red glow indicates that this region has a positive influence on the property (i.e. the property value increases) while a blue glow indicates a negative influence with green representing no significant overall effect. Unfortunately, a detailed description of the algorithm used for the Glowing Molecule method were not provided and, since it is implemented as part of a commercial product, the method is not generally available.

Here, we present similarity maps, a general approach for the visualization of both fingerprint similarities between two molecules and machine-learning (ML) model predictions. In our scheme, the "weight" of an atom is the similarity or predicted-probability difference obtained when the bits in the fingerprint corresponding to the atom are removed, similar to the approach of Franke *et al.*[6]. The normalized weights are then used to color the atoms in a topography-like map with green indicating a positive difference (i.e. the similarity or probability decreases when the bits are removed) and pink indicating a negative difference, gray represents no change. The visualization is demonstrated for atom pairs and several types of circular fingerprints and subsequently used to explain the factors leading to the predicted probability of a random forest and a naïve Bayes model. All source code and data required to reproduce the examples is provided in the Additional file 1.

## Implementation

A "weight" is determined for each atom of the test molecule by removing the bits which are set by the atom in the fingerprint of the test molecule, recalculating the similarity between the modified fingerprint and the fingerprint of the reference compound *s*_*mod*_, and calculating the difference to the original similarity Δ*s* = *s*_*orig*_ - *s*_*mod*_. The fingerprints are calculated using the open-source cheminformatics toolkit RDKit [10]. Dice [2] similarity is used in the current implementation but any other similarity metric could be employed. For AP (a count vector), the bits of an atom *i* are straightforward to determine, the count for each pair involving atom *i* is decreased by one. In circular fingerprints, on the other hand, bits are set for different atomic environments, starting at radius 0 up to the maximum radius. In RDKit, the environment (i.e. centre atom and radius) associated with each bit in a fingerprint can be obtained when generating the fingerprint. This information is used to determine all the bits where the atom is part of the environment.

The procedure to calculate "atomic weights" for the similarity between two molecules *ref_mol* and *this_mol* is shown in pseudocode below,

Similarity maps can also be used to visualize the atomic contributions to the predicted probability of a ML model. The generation of the bitmap is the same as before, depending on the kind of basic fingerprint used to train the ML model. However, the "atomic weights" are no longer similarity differences but predicted-probability differences,

In the case of NB, the difference between the logarithmic probabilities is used. The ML methods were calculated using the open-source toolkit scikit-learn [11].

To construct a similarity map, the atom weights are normalized by dividing by the maximum absolute weight value and then used to calculate bivariate Gaussian distributions centered at the corresponding atom positions. The atom weights influence only the peak and not the variance of the Gaussian distribution. The RDKit function for this makes use of the Python library matplotlib [12]. The similarity map is then generated by superimposing the atom coordinates with the Gaussian distributions and the contours using a matplotlib figure.

## Results and discussion

**Figure 1:**
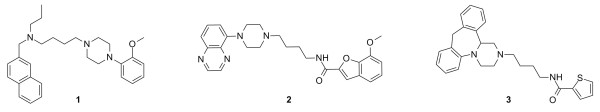
**Three dopamine D3 receptor ligands.** Reference compound **1** and test molecules **2** and **3**.

The use of similarity maps is demonstrated using ligands of the dopamine D3 receptor. The D3 receptor is one of five subtypes that belong to the G protein-coupled receptor (GPCR) superfamily. D3 receptor ligands contain a positively charged group, usually a protonatable tertiary amine, which forms a structurally and pharmacologically critical salt bridge to the carboxylate of Asp110^3.32^ as found by site-directed mutagenesis [13] and confirmed by the crystal structure [14]. Asp110^3.32^ is highly conserved in all aminergic receptors. Three active molecules (activity smaller than 10 *μ*M) of the D3 receptor (ChEMBL [15,16] target ID 130) from three different scientific papers [17-19] were extracted from the ChEMBL database (Figure 1). Molecule **1** was selected as reference compound and the other two as test molecules.

### Standard fingerprints

The similarity between the reference compound **1** and the test molecules was calculated using four different 2D fingerprints: atom pairs (AP) [20], circular fingerprint [21] with radius 2 as bit vector (Morgan2) and as count vector (CountMorgan2), and feature-based circular fingerprint [21] with radius 2 as bit vector (FeatMorgan2). The fingerprints are described in detail in [22]. Morgan2 is the RDKit implementation of the familiar ECFP4, CountMorgan2 corresponds to ECFC4 and FeatMorgan2 to FCFP4 [23]. The features used by the RDKit for FeatMorgan2 are adapted from [24] and consist of donors, acceptors, aromatic atoms, halogens, basic and acidic atoms. The numerical similarity and maximum differences obtained for the four fingerprints are given in Table 1.

**Table 1:** Dice similarities and maximum weights

**FP**	**s**_**Dice**_		**Max. Weight**		**Method**	**PP**		**Max. Weight**	
	**2**	**3**	**2**	**3**		**2**	**3**	**2**	**3**
AP	0.604	0.531	0.028	0.033	RF(Morgan2)	0.950	0.600	0.660	0.370
Morgan2	0.561	0.381	0.123	0.110	NB(Morgan2)	1.000	0.999	12.5	20.92
CountMorgan2	0.599	0.529	0.091	0.049					
FeatMorgan2	0.554	0.469	0.176	0.171					

Dice similarity *s*_*Dice*_ and maximum weight between reference compound **1** and molecules **2** and **3** using the basic fingerprints (FP) atom pairs (AP), Morgan fingerprint as bit vector with radius 2 (Morgan2), Morgan fingerprint as count vector with radius 2 (CountMorgan2) and Feature Morgan fingerprint with radius 2 (FeatMorgan2). Predicted probability to be active (PP) and maximum weight for random forest (RF) and naïve Bayes (NB) for molecules **2** and **3** with basic fingerprint Morgan2. The bit vectors of the circular fingerprints had the size 1024 bits. The default maximum path length of 30 was used for AP.

**Figure 2:**
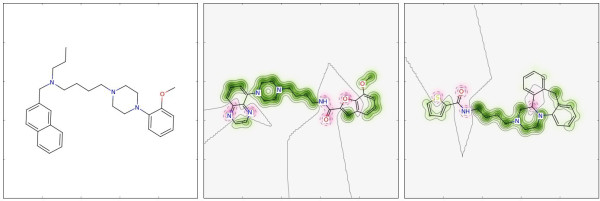
**Similarity maps for atom-pairs (AP) fingerprint.** Similarity map of molecule **2** (middle) and molecule **3** (right) using AP. The reference compound is molecule **1** (left). Color scheme: removing bits decreases similarity (i.e. positive difference) (green), no change in similarity (gray), removing bits increases similarity (i.e. negative difference) (pink). The default maximum path length of 30 was used for AP.

The similarity maps of molecules **2** and **3** using the AP fingerprint are shown in Figure 2. An atom in the AP fingerprint sees all other atoms (if the path is maximum 30 bonds). Atoms with green weights have a majority of paths which are also in the reference compound; deleting them from the fingerprint reduces the similarity to the reference compound. The similarity maps in Figure 2 are consistent with our expectations. For molecule **2**, atoms in the phenyl rings, the piperazine moiety and the alkyl linker were found important for similarity, whereas removing the bits of the nitrogens in the quinoxaline moiety, the oxygen in the benzofuran moiety, or the amide increased the similarity. Also for molecule **3**, atoms in the alkyl linker and partly in the piperazine moiety were found to be most important for similarity.

**Figure 3:**
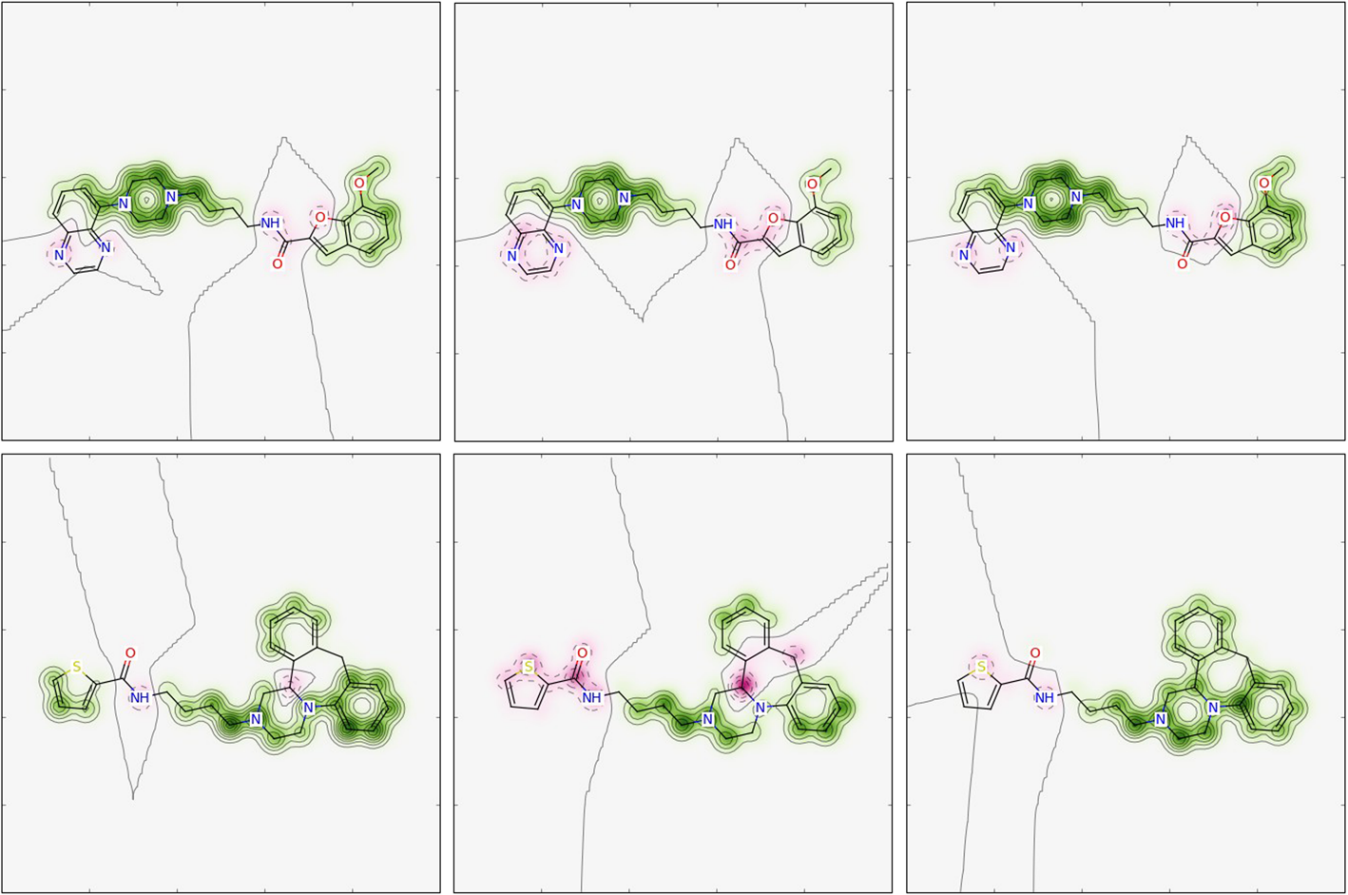
**Similarity maps for circular fingerprints.** Similarity map of molecule **2** (middle) and molecule **3** (bottom) using Morgan2 (left), CountMorgan2 (middle) and FeatMorgan2 (right). The reference compound is molecule **1** (left panel in Figure 2). Color scheme: removing bits decreases similarity (i.e. positive difference) (green), no change in similarity (gray), removing bits increases similarity (i.e. negative difference) (pink). The bit vectors of the circular fingerprints had the size 1024 bits.

**Figure 4:**
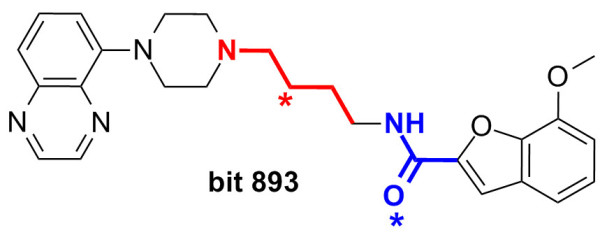
**Bit collision in Morgan2/CountMorgan2.** Bit Collision in the Morgan2 and CountMorgan2 fingerprint observed for molecule **2** (and analoguously in molecule **3**). The environments highlighted red and blue are hashed to the same bit. The centre atom of an environment is marked with a star.

The similarity maps of the circular fingerprints, Morgan2, CountMorgan2 and FeatMorgan2, are shown in Figure 3. In circular fingerprints, an atom sees only a local environment. Again, the piperazine moiety together with the alkyl linker as well as part of the 7-methoxybenzofuran are highlighted green in molecule **2** for all three variants of the circular fingerprint. Interestingly, the pyrazine part of quinoxaline and the amide appear more pink for CountMorgan2 than for Morgan2. In the first case, one can observe the difference between using a count vector and a bit vector. Using CountMorgan2, the count of the radius-0 bit of the unsubstituted carbons of the pyrazine moiety is 11 for the reference compound and nine for molecule **2**, the count of the radius-1 bit is zero and two. Using Morgan2, the radius-0 bit is set to one in both molecules, whereas the radius-1 bit is zero in the reference compound and one in molecule **2**. Removing the radius-1 bit or decreasing its count will increase the similarity. Removing the radius-0 bit will decrease the similarity, whereas decreasing its count from nine to eight will only have a very small effect on similarity. Thus, the overall "atomic weight" of these carbons is negative (pink) for CountMorgan2, but neutral for Morgan2. The reason for the different appearance of the amide bond, on the other hand, is a hash collision (Figure 4) in the Morgan2 fingerprint: an environment of the amide moiety is hashed to the same bit as a part of the alkyl linker. The same effect can be observed for molecule **3**. This collision appears only in Morgan2, which is hashed to a size of 2^10^ bits whereas CountMorgan2 uses 2^32^ bits. It is generally important to use a sufficiently large hash space as collisions can impact the performance of a fingerprint [25]. However, the occurrence of collisions is also dependent on the hashing algorithm used. For Morgan2, increasing the bit-vector size from 2^10^ bits to 2^14^ bits had no influence on the performance [22], and also in the current case doubling the hash space (i.e. 2^11^ bits) did not remove the observed collision (data not shown).

The features in the reference compound are aromatic rings, two acceptors and two basic acceptors. These features are marked green in the right panels in Figure 3 for both molecules. Removing the aromatic acceptors or the donor in the molecules, on the other hand, increased the similarity to the reference compound. Interestingly, one carbon of the piperazine moiety in molecule **3** is highlighted pink using CountMorgan2 (and to a lesser extent using Morgan2) whereas it is green using FeatMorgan2. For (Count)Morgan2, the atom type of this carbon is different than the atom types of the other carbons as the number of heavy-atom neighbours and the number of hydrogens is different. Using features (donor, acceptor, aromatic, basic, acidic, no-feature), however, the number of neighbours and hydrogens are not considered, thus the feature type (i.e. no-feature) is the same for all carbons in the piperazine.

### Machine-learning methods

Two kinds of machine-learning (ML) methods, random forest (RF) and naïve Bayes (NB), were trained and used to predict the probability to be active of new molecules. The reference compound and the other active molecules (activity smaller than 10 *μ*M) from Ref. [17] (Figure S1 in Additional file 2) were used together with randomly selected 10% of the 10000 ChEMBL decoys used in a recent benchmarking study [22] to train the ML models. Morgan2 was used as the standard fingerprint. The following optimal parameters of random forests have been determined through a grid search: number of trees (*N*_*T*_) = 100, maximum depth = 2, minimum samples to split = 2 and minimum samples per leaf = 1. To avoid the problems caused by imbalance in the training set (i.e. many more inactives than actives) for RFs, the balanced random forest algorithm [26] was applied: for each decision tree the majority class is down-sampled to yield an equal number of instances as the minority class. The naïve Bayes classifier was trained using an additive Laplace smoothing parameter of 1.0 and learned class prior probabilities.

**Figure 5:**
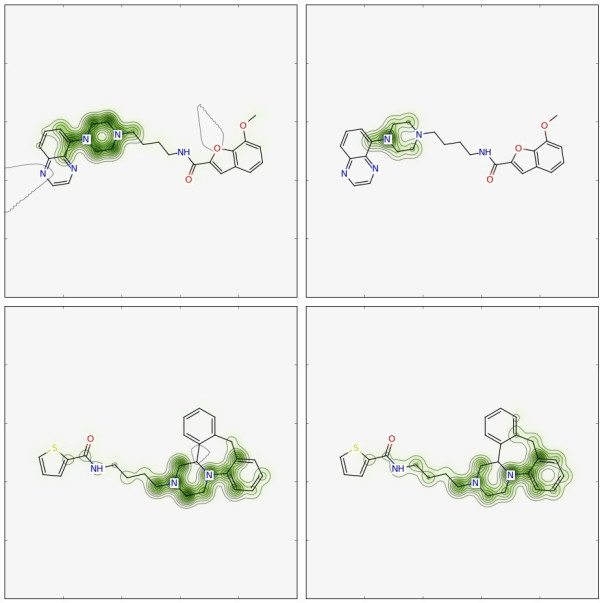
**Similarity maps for machine-learning methods.** Similarity map of molecule **2** (top) and molecule **3** (bottom) using RF(Morgan2) (left) and NB(Morgan2) (right). Color scheme: removing bits decreases similarity (i.e. positive difference) (green), no change in similarity (gray), removing bits increases similarity (i.e. negative difference) (pink). The bit-vector size of Morgan2 was 1024 bits.

The similarity maps (or predicted probability maps, respectively) for the RF model trained with Morgan2 are shown in the left panels of Figure 5. For both molecules, the RF picked up the piperazine moiety with the attached alkyl chain and part of the aromatic fragment. Looking at the active molecules of Ref. [17] (Figure S1 in Additional file 2) confirms that the aromatic ring - piperazine - alkyl chain motif appears in the vast majority of active compounds. Thus, the RF model was able to extract the important structural feature for activity: the nitrogen in the piperazine moiety is protonated at physiological pH and forms the critical salt bridge with Asp110^3.32^ of the receptor [13,14].

Similar findings were obtained for the NB model (right panels in Figure 5). Again, the piperazine moiety was found to be most important.

## Conclusions

Similarity maps are an easy and general strategy for the visualization of the atomic origins of fingerprint similarity between molecules. The "atomic weights" are generated by removing the bits belonging to the corresponding atom and comparing the resulting similarity with the similarity of the unmodified fingerprint. Similarity maps can be generated for every fingerprint that allows a backtracking of the bits to a corresponding atom or substructure. The methodology can be extended to machine-learning (ML) models to visualize the atomic contributions to the predicted probability of the ML model. This is especially useful as ML models often appear as black boxes. In future work, we will investigate the application of the visualization strategy to descriptor-based models for physicochemical-property prediction.

## Availability and requirements

The source code is provided in Additional file 1. The implementation used the open-source Python toolkits RDKit [10] version 2013.03, scikit-learn [11] version 0.13, and matplotlib [12] version 1.1.0.

## Competing interests

The authors declare that they have no competing interests.

## Authors’ contributions

SR participated in the conception of the visualization approach, collected the data sets, developed and generated the similarity maps, and drafted the manuscript. GL participated in the conception of the visualization approach and in the discussion of the results, and helped to draft the manuscript. Both authors read and approved the final manuscript.

## Supplementary Material

Additional file 1 **Source Code.** The file python_scripts.zip contains the source code of the visualization method and the SMILES of the compounds used to generate the figures in the publication. Click here for file

Additional file 2 **Supplementary Figures and Tables.** The file supplementary.pdf contains the additional figure mentioned in the text. Click here for file
