# Supplementary material for: Similarity maps - a visualization strategy for molecular fingerprints and machine-learning methods
Source: J Cheminform. 2013 Sep 24;5:43. doi: 10.1186/1758-2946-5-43 (PMC3852750; doi:10.1186/1758-2946-5-43)
Supplement: Additional file 2 — Supplementary Figures and Tables. The file supplementary.pdf contains the additional figure mentioned in the text. [file 1758-2946-5-43-S2.pdf]

Open-Source Platform to Benchmark Fingerprints for Ligand-Based Virtual Screening

Sereina Riniker[1] and Gregory A. Landrum*[1]

[1] Novartis Institutes for BioMedical Research, Basel, Switzerland

Email: Sereina Riniker - sereina.riniker@novartis.com; Gregory A. Landrum* - gregory.landrum@novartis.com;

*Corresponding author

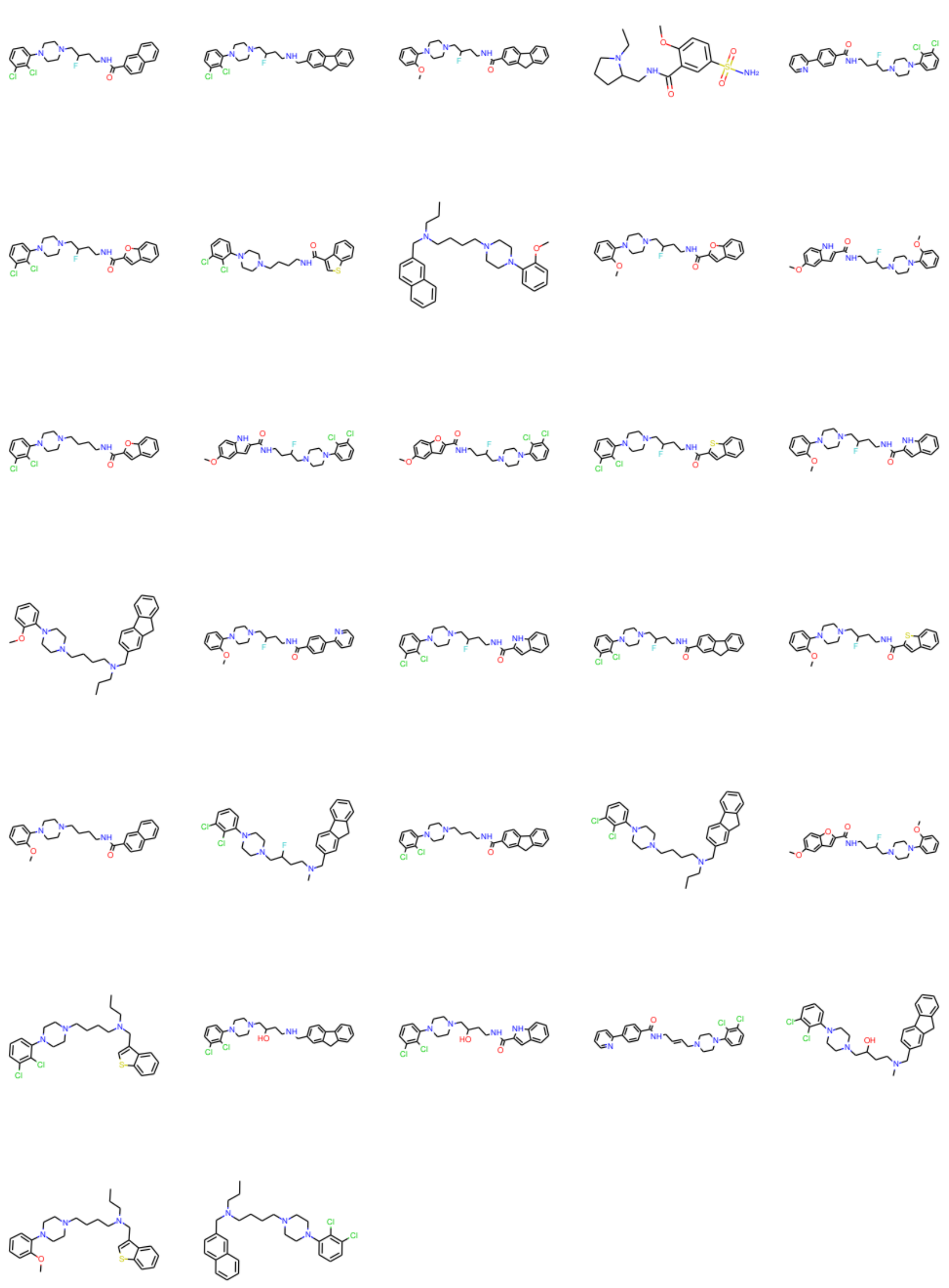

Figure S1: Active molecules (activity smaller than 10 μM) of [1]

References

1. Banala AK, Levy BA, Khatri SS, Furman CA, Roof RA, Mishra Y, Griffin SA, Sibley DR, Luedtke RR, Newman AH: ***N*-(3-Fluoro-4-(4-(2-methoxy or 2,3-dichlorophenyl)piperazine-1-yl)butyl)arylcarboxamides as selective dopamine D3 receptor ligands: critical role of the carboxamide linker for D3 receptor selectivity**. *J. Med. Chem.* 2011, **54**:3581–3594.
